# Gastric Epithelioid Angiosarcoma: An Unexpected Tumor in an Unexpected Location

**DOI:** 10.7759/cureus.15049

**Published:** 2021-05-15

**Authors:** Artem Sharko, Shirly Samuel, Grace W Ying, Sonika Prasad, Shaji Baig

**Affiliations:** 1 Internal Medicine, Northwestern Medicine McHenry Hospital, Rosalind Franklin University of Medicine and Science, McHenry, USA; 2 Internal Medicine, Chicago Medical School Internal Medicine Residency Program at Northwestern Medicine McHenry Hospital, McHenry, USA

**Keywords:** epithelioid angiosarcoma, acute gastrointestinal bleed, gastric angiosarcoma, gastrointestinal tumors, gastric polyps

## Abstract

Angiosarcomas are aggressive neoplasms that arise from endothelial cells and can develop in any part of the body. Gastrointestinal angiosarcomas are very uncommon and can have a variable clinical presentation. We report a case of an 84-year-old female who presented with acute blood loss anemia. She underwent a gastrointestinal workup with esophagogastroduodenoscopy (EGD), which revealed two polyps in the stomach. Histologic evaluation of the polyps was indicative of angiosarcoma, and the diagnosis was eventually confirmed by immunohistochemical analysis with positive CD31, ERG, and FLI1 stains. This case is reported to demonstrate the importance of considering angiosarcoma in the differential for patients presenting with gastrointestinal bleeding.

## Introduction

Angiosarcoma is a form of malignant tumor that arises from the endothelium of blood vessels. These tumors can occur in various sites in the body and can present with a variety of clinical pictures depending on their location. They are incredibly aggressive, can metastasize early, and have a poor prognosis, especially if they are diagnosed late in the course of the disease. Because angiosarcomas are rare overall and are even more uncommon in the gastrointestinal tract, they are often overlooked in the differential diagnosis of gastrointestinal tumors. The main risk factor for the development of angiosarcomas appears to be radiation. With radiation being used increasingly more over the last decades, it is reasonable to expect a rise in the incidence of angiosarcomas in the near future. Moreover, with the disease being so infrequently encountered, the diagnosis can be easily missed, leading to poor outcomes [1]. We present an extremely rare case of gastric epithelioid angiosarcoma with the goal to raise awareness of this disease. We describe the pathological features of epithelioid angiosarcoma and its presentation as an upper gastrointestinal bleed in an 84-year-old female who presented with an acute drop in her hemoglobin.

## Case presentation

An 84-year-old Caucasian female presented with an acute drop in hemoglobin from 7.9 g/dL to 6.9 g/dL, noted during a routine laboratory investigation. The patient's baseline hemoglobin was 8-10 g/dL. On presentation, the patient endorsed fatigue, lasting about three months, as well as shortness of breath on exertion. The patient denied fever, chills, night sweats, unintentional weight loss, abdominal pain, nausea, vomiting, diarrhea, constipation, hematochezia, hematemesis, or hemoptysis. Two years prior, she was diagnosed with iron deficiency anemia and was managed with iron supplements by her hematologist. At that time, she also underwent a workup to rule out a gastrointestinal bleed with EGD and colonoscopy, which revealed mild chronic reactive gastritis with erosions and grade II internal hemorrhoids. Her past medical history also included atrial fibrillation, gastroesophageal reflux disease, essential hypertension, hyperlipidemia, a cerebral vascular accident, and vascular dementia. Surgical history was negative for any gastrointestinal procedures. Her medications included rivaroxaban, omeprazole, metoprolol, and atorvastatin. She had never smoked cigarettes and drank alcohol socially. There was no history of any gastrointestinal malignancies, inflammatory bowel diseases, or other gastrointestinal illnesses in the family.

**Figure 1:**
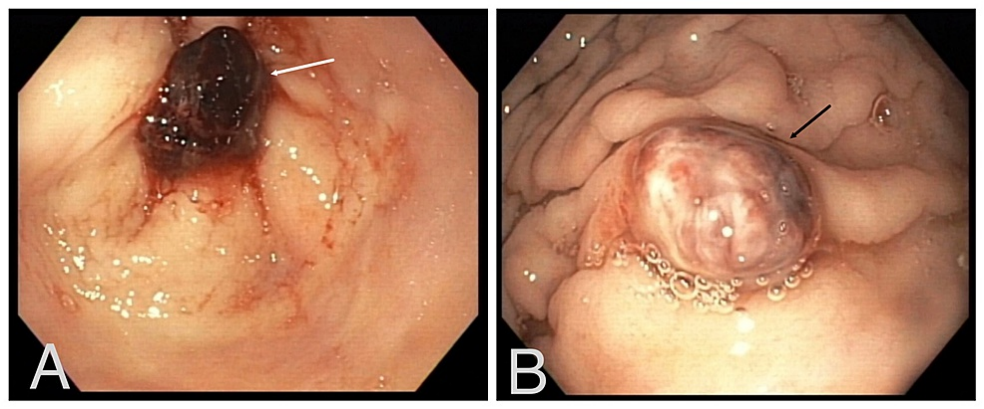
EGD: Hemorrhagic polyp in the pyloric channel of the stomach (A: white arrow). Second polyp in the fundus of the stomach (B: black arrow).

In the emergency department, her blood pressure was 117/66 mmHg, heart rate 75 beats per minute, temperature 37 °C, respiratory rate 20 breaths per minute, and oxygen saturation 95% on room air. On physical examination, the patient appeared pale but was not in acute distress. The abdominal exam revealed a soft, non-distended, non-tender abdomen with no palpable masses. The rectal exam was positive for melenic stool. A complete blood count revealed hemoglobin of 6.6 g/dL (reference range, 12-16 g/dL), hematocrit of 21.3% (reference range, 36-46%), and mean corpuscular volume of 104.7 fL (reference range, 90-99 fL). A peripheral blood smear reported macrocytic anemia with mild anisocytosis and polychromasia. The vitamin B12 level was 976 pg/mL (reference range, 220-1100 pg/mL) and the folate level was 23.3 mg/mL (reference range, >5.3 ng/mL). Other laboratory findings, including white blood cell count, platelets, electrolytes, blood urea nitrogen, creatinine, and liver function tests, were all within normal ranges. The patient underwent a blood transfusion with one unit of packed red blood cells. She received a bolus of intravenous pantoprazole, followed by an infusion of the same. Her home medication of rivaroxaban was held on admission. The following day, her hemoglobin and hematocrit improved to 9.2 g/dL and 26%, respectively. The patient did not have any bowel movements. An EGD was performed and revealed a large oozing hemorrhagic polyp in the pyloric channel and a second similar, smaller polyp in the fundus (Figure 1). The bases of the polyps were injected with epinephrine, and snare cautery was used to remove them, followed by placement of clips over the bases for hemostasis. No evidence of gastritis or esophagitis was seen. Given the high risk of bleeding, the patient was advised to stay off anticoagulation for five days. Her hemoglobin continued to trend up, and she did not have any more episodes of melena. The patient was discharged in stable condition with pathology evaluation of the polyps pending and instructions to follow up with her gastroenterologist and oncologist.

**Figure 2:**
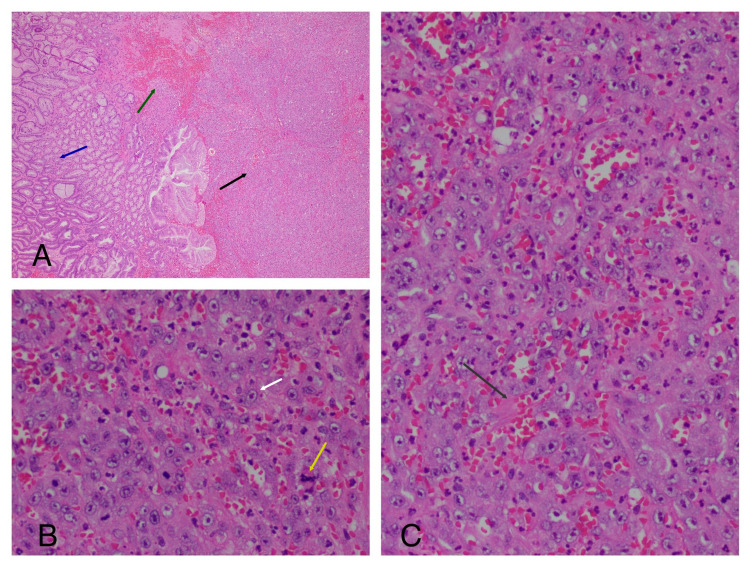
Hematoxylin and eosin stain: (A) On the slide, we can see normal pyloric mucosa (blue arrow), the tumor (black arrow), and areas of hemorrhage (green arrow). (B) The tumor is composed of a predominantly solid distribution of moderately atypical, large epithelioid cells with ovoid vesicular nuclei and prominent nucleoli (white arrow). We can see mitotic figures (yellow arrow). The mitotic activity is high with 2-3 mitoses/HPF. (C) Poorly formed vascular spaces lined by atypical endothelial cells are also noted. The neoplasm is associate with extensive hemorrhage (grey arrow).

**Figure 3:**
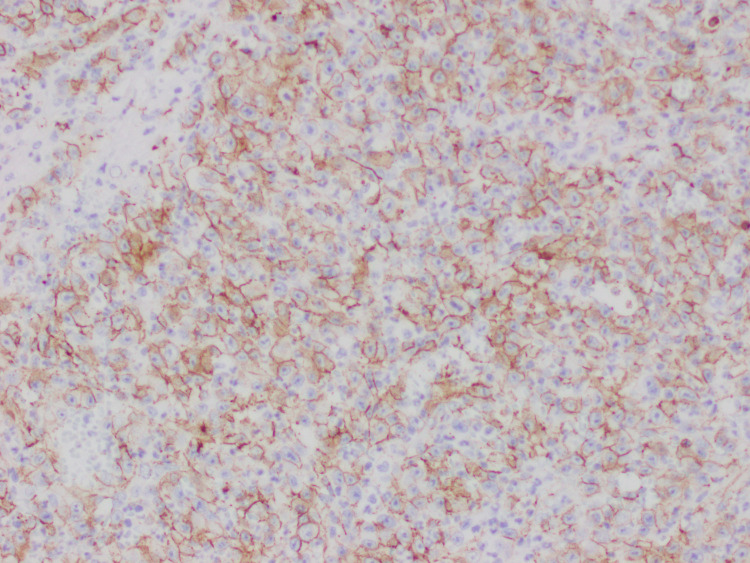
Immunohistochemical stain positive for CD 31.

**Figure 4:**
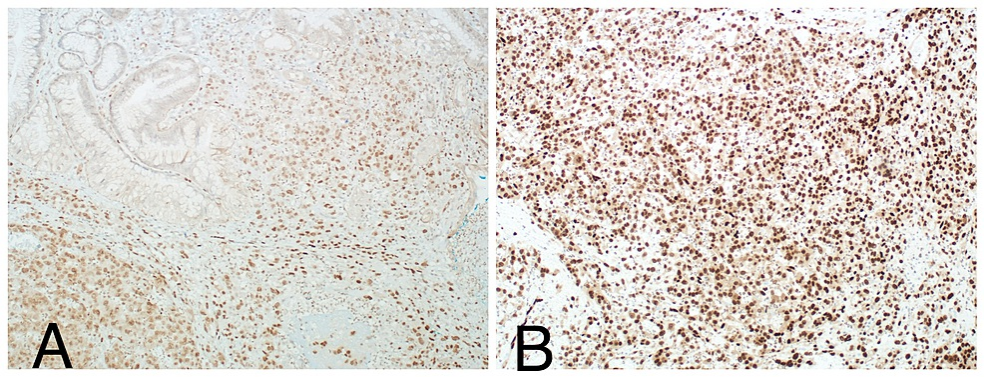
Immunohistochemical stains: positive for FLI1 (A) and ERG (B).

**Figure 5:**
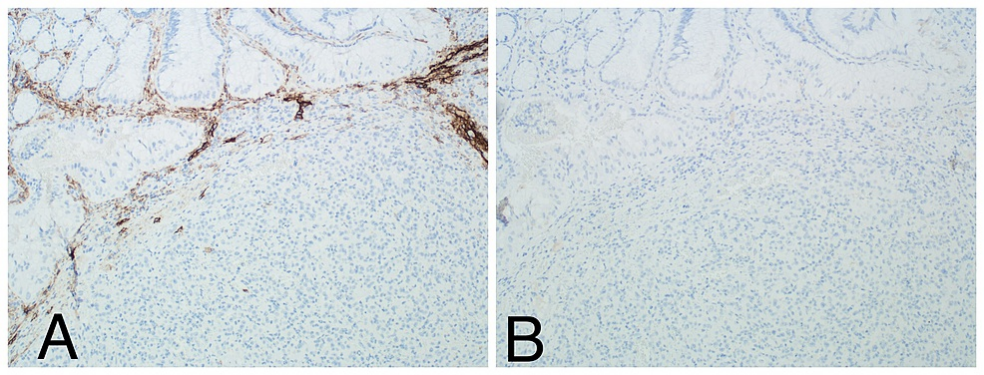
Immunohistochemical stains: negative for D2-40 (A) and LANA (B).

Pathology reported two dark red polyps measuring 0.7 cm and 1.2 cm in their greatest dimension. Histologic analysis of the polyps showed that the tumors consisted of moderately atypical, large epithelioid cells with ovoid vesicular nuclei and prominent nucleoli. The neoplasm was associated with extensive hemorrhage and high mitotic activity (Figure 2). The diagnosis of angiosarcoma was supported by a positive stain for vimentin, vascular markers, such as CD31 (Figure 3), ERG, and FLI1 (Figure 4), and epithelial markers, such as keratin AE1/AE3 and CK8/18. Immunohistochemical stains for LANA and D2-40 (Figure 5), as well as Melan A, SOX10, S100, HMB45, MITF, SMA, CD117, and DOG1 were negative.

 

 

 

## Discussion

Angiosarcomas are a rare form of malignant connective tissue neoplasms that arise from the endothelial cells of blood vessels. These tumors are known for their rapid proliferation, aggressive infiltration, hematogenous metastasis, and relatively poor prognosis with low life expectancy. Depending on the primary lesion site, they can metastasize to various organs in the body, including, but not limited, to lymph nodes, liver, lungs, and brain [2]. The mean age of diagnosis is reported to be 65 [2]. The neoplasm has the ability to arise in any part of the body, with approximately 60%-70% of the cases being seen on the skin of the head and neck, and with the majority of the remaining cases being reported in the heart, kidneys, and lungs. To a rare extent, the gastrointestinal tract is another site where angiosarcomas can occur. In these cases, the spleen and liver are the most commonly associated organs, with only a few cases reporting colonic and intestinal involvement [3]. Gastric angiosarcomas, however, appear to be extremely rare, and to the best of our knowledge, only two cases have been reported thus far [2,4]. Due to the multitude of organs that angiosarcomas can arise from, differentiating between a primary and a metastatic lesion can be a difficult task and is not always achievable [3].

Studies have shown that radiation, especially when it leads to subsequent lymphedema, known as Stewart-Treves syndrome, can lead to the development of angiosarcomas. Moreover, environmental factors such as exposure to arsenic or vinyl chloride, as well as genetic syndromes such as xeroderma pigmentosa, bilateral retinoblastoma, and von Recklinghausen neurofibromatosis are also known to be significant risk factors [1,5].

Angiosarcomas can present in various ways depending on their primary location. All lesions, however, are prone to ulceration and bleeding, which is especially important in patients with intestinal angiosarcomas whose only presenting symptom may be anemia. Other reported symptoms with intestinal involvement may include generalized abdominal pain or discomfort, nausea, vomiting, diarrhea, or symptoms suggestive of luminal obstruction in cases of rapid cellular growth [6].

Imaging studies such as computed tomography and magnetic resonance imaging can reveal and localize lesions in the GI tract. Other forms of imaging studies, such as positron-emission tomography, are useful for disease staging; however, they lack the ability to differentiate between different types of tumors [2]. The only way to definitively diagnose an epithelioid angiosarcoma is by immunohistochemical analysis. CD31 is one of the most reliable markers [2]. Studies have shown that stains for CD31 and ERG are very reliable and have close to 100% sensitivity for angiosarcomas [7,8]. When both ERG and CD31 are positive, the diagnosis of angiosarcoma can be made with high confidence. Other markers such as vimentin, vascular (FLI1, Factor VIII), and epithelial (cytokeratin AE1/AE3, CK8/18) are also used but are less sensitive. The diagnosis can also be supported by negative staining for LANA and D2-40, which are highly sensitive for Kaposi sarcoma [7]. In our patient, the diagnosis of angiosarcoma was confirmed by a positive stain for vimentin, vascular (ERG, FLI1, CD31), and epithelial (keratin AE1/AE3, CK8/18) markers in tumor cells. Cells stained negative for LANA, D2-40, Melan A, SOX10, S100, HMB45, MITF, SMA, CD117, and DOG1, thus excluding Kaposi sarcoma, melanoma, angiomyolipoma, and gastrointestinal stromal tumor [9].

Histologically, angiosarcoma may range from a presentation of vascular proliferation in less malignant forms to an undifferentiated tumor in malignant cases. It is characterized by spindled, polygonal, or epithelioid cells with nuclear division and pathological mitotic figures [2,5]. The presence of epithelioid endothelial cells and cavernous vessels helps differentiate angiosarcomas from Kaposi sarcoma, which on the other hand, has eosinophilic hyaline granules and stains positive for smooth muscle actin [6]. Macroscopically angiosarcoma can be categorized as mucosal or submucosal. Mucosal lesions can be obtained for analysis with endoscopic biopsy. Submucosal lesions, however, usually require an ultrasound-guided endoscopic procedure to obtain the specimen for pathology evaluation [3].

The primary treatment modality of angiosarcoma is surgical clean-margin excision, which in some cases can be curative. Radiotherapy is often used as adjuvant treatment, even when margins are clean, to decrease recurrence. It may be used as the main management option in non-resectable tumors [1]. Of note, radiation therapy for angiosarcoma may itself lead to secondary angiosarcomas. Chemotherapy drugs such as paclitaxel and doxorubicin are used in cases of metastatic angiosarcoma; however, they appear to have little, if any, benefit as adjuvant treatment after surgery and radiation [5]. They are also an option for non-resectable tumors as part of palliative care or for converting the tumor to resectable [1]. Paclitaxel has shown promising results in phase II of a clinical trial for the treatment of non-resectable breast angiosarcoma [10]. VEGF receptor inhibitors such as bevacizumab and tyrosine kinase inhibitors such as sorafenib are possible treatment options for metastatic angiosarcomas; however, current studies have not shown significant effect so far [5,11]. Immunotherapy, including antibodies against programmed cell death protein 1 (PD-1) and programmed death-ligand 1 and 2 (PD-L 1 and PD-L 2), have shown promising results. Specifically, pembrolizumab, a PD-L 1 inhibitor, has been shown to improve the prognosis in angiosarcoma patients [5,12]. We still, however, need larger research studies to make definitive conclusions about the effectiveness of this group of medications [5].

The prognosis for patients diagnosed with angiosarcoma is very poor, and it is determined by a number of factors. Different studies have shown that the overall survival at five years since diagnosis is estimated to be 31%-43%, with a median survival time of approximately 16-42 months [1,2,13]. The life expectancy for patients with metastatic tumors is about 3-12 months [1,13]. Finally, patients with angiosarcoma of the gastrointestinal tract usually have a much worse prognosis, with the majority dying within a year after initial diagnosis [14]. Negative prognostic factors include metastatic disease, the size of the primary tumor being 5 cm or more, and the tumor's location being deep tissue or internal organ [1].

## Conclusions

This case is presented to raise awareness of angiosarcoma as a possible source of gastrointestinal bleeding. Our patient lacked any obvious risk factors for angiosarcoma, and thus without relevant immunohistochemical staining, her lesion could have been interpreted as Kaposi sarcoma, leiomyoma, gastrointestinal stromal tumor, or a Dieulafoy's lesion. Such misdiagnoses have been reported in the past, which can be explained by the fact that gastrointestinal angiosarcomas can present with various macroscopic morphologies such as ulcers, submucosal nodules, or hemorrhagic lesions. A delay in diagnosis could lead to potentially preventable recurrence and metastasis of the tumor, which can have catastrophic consequences for the patient. We believe it is essential to consider angiosarcoma as part of the differential of gastrointestinal bleeding. Additionally, screening for risk factors for angiosarcoma can help to expedite the diagnostic process leading to improved prognosis and outcomes for the patients.
